# Management of COVID-19-related post-intubation tracheal stenosis

**DOI:** 10.3389/fsurg.2023.1129803

**Published:** 2023-03-09

**Authors:** Serena Conforti, Gloria Licchetta, Marco Reda, Arash Astaneh, Luca Pogliani, Stefano Fieschi, Alessandro Rinaldo, Massimo Torre

**Affiliations:** Department of Thoracic Surgery, ASST Grande Ospedale Metropolitano Niguarda, Milano, Italy

**Keywords:** COVID-19, tracheal stenosis, endoscopic treatment, tracheal surgery, balloon dilatation

## Abstract

**Introduction:**

The Severe Acute Respiratory Syndrome Coronavirus 2 (SARS-CoV-2) pandemic has affected Italy since the beginning of 2020. Endotracheal intubation, prolonged mechanical ventilation, and tracheostomy are frequently required in patients with severe COVID-19. Tracheal stenosis is a potentially severe condition that can occur as a complication after intubation. The aim of this study was to evaluate the utility and safety of endoscopic and surgical techniques in the treatment of tracheal stenosis related to COVID-19.

**Materials and Methods:**

Between June 2020 and May 2022, consecutive patients with tracheal stenosis who were admitted to our surgical department were considered eligible for participation in the study.

**Results:**

A total of 13 patients were included in the study. They consisted of nine women (69%) and four men (31%) with a median age of 57.2 years. We included seven patients with post-tracheostomy tracheal stenosis. Bronchoscopy was performed to identify the type, location, and severity of the stenosis. All patients underwent bronchoscopic dilation and surveillance bronchoscopy at 7 and 30 days after the procedure. We repeated endoscopic treatment in eight patients. Three patients underwent tracheal resection anastomosis. Final follow-up bronchoscopy demonstrated no residual stenosis.

**Conclusions:**

The incidence of and risk factors associated with tracheal stenosis in critically ill patients with COVID-19 are currently unknown. Our experience confirms the efficacy and safety of endoscopic management followed by surgical procedures in cases of relapsed tracheal stenosis.

## Introduction

Over the last few years, the world has been hit by pandemic waves of a new coronavirus known as Severe Acute Respiratory Syndrome Coronavirus 2 (SARS-CoV-2), which has caused >90 million infections (1). From January 2020, the World Health Organization (WHO) considered this disease to be a public health emergency (2). This infection has a wide variety of clinical presentations, ranging from asymptomatic to severe cases of acute respiratory distress syndrome (ARDS) (3). Prior to the pandemic, up to 9% of patients requiring invasive ventilation experienced tracheal stenosis (6). During the COVID-19 era, this rate increased. Up to 90% of patients admitted to an intensive care unit (ICU) undergo intubation and invasive mechanical ventilation, often requiring tracheostomy (4). COVID-19 patients have a median ventilation duration of 17 days and a high frequency of reintubation (5). As reported in the literature (6), prolonged mechanical intubation may lead to mucosal damage and inflammation, the development of granulation tissue, and the subsequent formation of cicatricial stenotic tissue (7, 8). In addition, prone position, overinflation of the tube cuff, and use of a larger endotracheal tube can contribute to the risk of stenosis (9).

**Figure 1:**
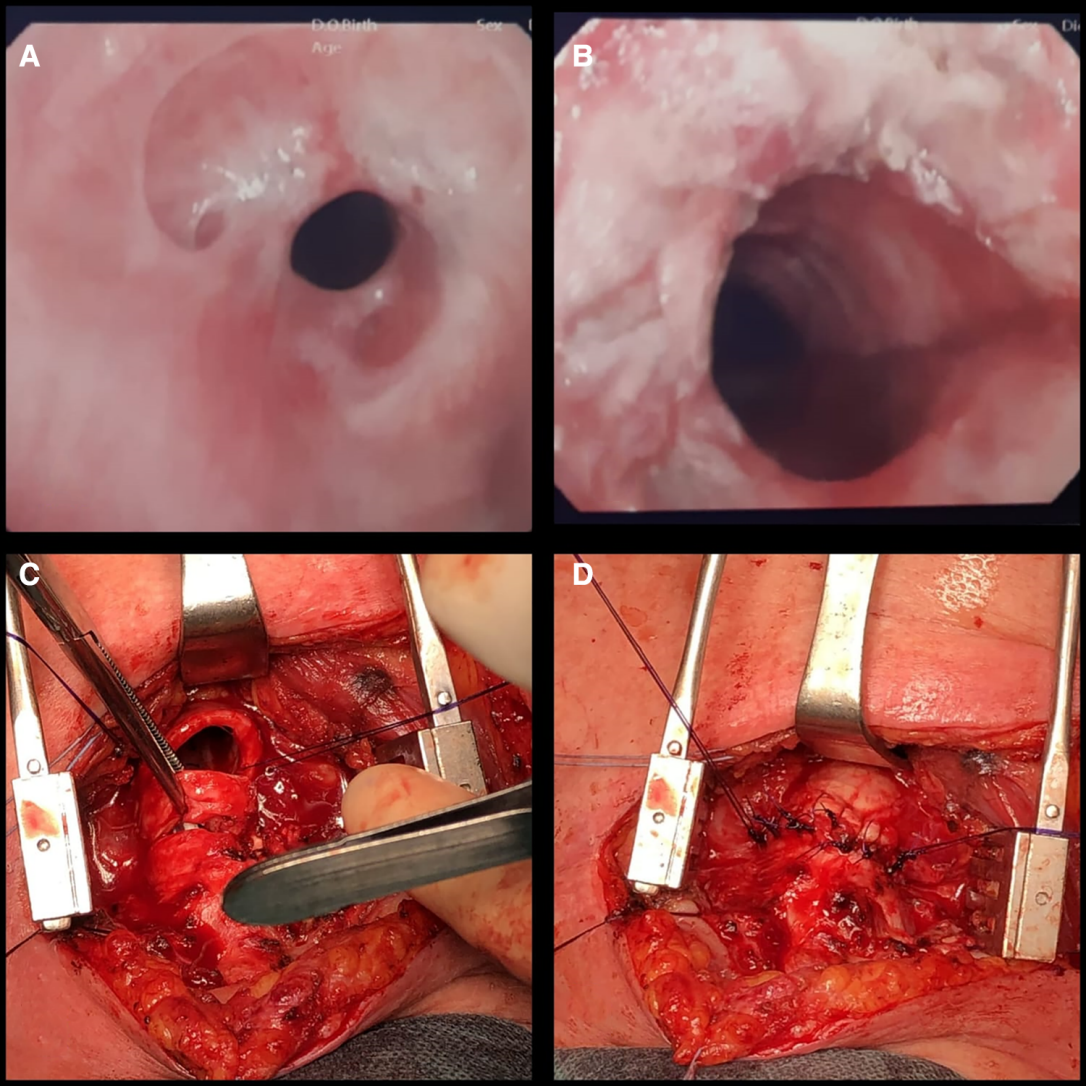
(**A**) Tracheal stenosis (Myer–Cotton grade II); bronchoscopic examination. (**B**) Endoscopic result after balloon dilation. (**C**) Surgical resection of the trachea. (**D**) Suture of the anterior tracheal wall.

Tracheal stenosis (TS) is usually the result of scar formation with associated morbidity depending on the location, extent, and thickness of the tissue. Tracheal stenosis can occur anywhere from the level of the endobronchial tube up to the glottic and subglottic area, particularly at the site where the tube cuff comes into contact with the tracheal mucosa and at the tracheal stoma site after the tracheostomy procedure is performed (10, 11). Multiple other factors create a predisposition to tracheal stenosis, such as a high tracheostomy site, traumatic intubation, infections, chronic inflammatory diseases, obesity, advanced age, excessive corticosteroid use, and autoimmune diseases. The symptoms are variable and depend on the site and grade of the stenosis (12). In terms of diagnosis, computerized tomography is used more often than magnetic resonance imaging and correlates well with endoscopic findings (13). However, bronchoscopy is the gold standard for diagnosis. This is performed to identify the type, location, and severity of the stenosis (11, 13) (Figure 1A). As regards management, we consider endoscopic and surgical approaches. Through endoscopic balloon dilation and intra-lesional corticosteroid injection, it is possible to guarantee a significant improvement in airway patency, avoiding tracheostomy. Endoscopic management does not preclude open surgical procedures, when necessary. The aim of this study was to evaluate the utility and safety of endoscopic and surgical techniques in the management of tracheal stenosis related to COVID-19.

## Methods

A retrospective, single-center series of cases was collected in an Italian training hospital. The need for informed consent from individual patients was waived owing to the retrospective nature of the study. All patients admitted between 1 June 2020 and 31 May 2022, inclusive of these dates, were screened for eligibility. The inclusion criterion was a laboratory-confirmed history of SARS-CoV-2 infection (i.e., positive result of real-time reverse transcriptase-polymerase chain reaction assay of nasal and pharyngeal swabs). The exclusion criteria were: age < 18 years; admission for causes other than respiratory failure; malignant or benign tracheal neoplasm; and idiopathic TS or other iatrogenic cases of tracheal stenosis. Clinical data were extracted from the patient data management system and a unique database was created. For every included patient, we recorded demographic and anthropometric data, comorbidities, and medical history. Information regarding airway management (i.e., performance of a tracheostomy, extubation, or decannulation) was acquired daily until ICU discharge. Data concerning treatments included the number and type of endoscopic therapeutic procedures. Finally, data concerning endoscopic and/or surgical outcomes and clinical follow-up duration were collected.

## Results

Between June 2020 and May 2022, 13 consecutive patients were admitted to our department for tracheal stenosis after COVID-19 infection. They consisted of nine women (69%) and four men (31%), with an age range of 45–72 years (median age: 57.2 years). Table 1 summarizes the baseline characteristics of the patients at ICU admission. Twelve patients (92.3%) had at least one comorbidity; specifically, eight (61.5%) presented with obesity (body mass index > 30 kg/m^2^), four (30.7%) with hypertension, five (38.4%) with diabetes mellitus, four (30.7%) with cigarette smoking and chronic obstructive pulmonary disease, and three (23%) with use of corticosteroids in autoimmune disease. Data regarding respiratory maintenance during COVID-19 consisted of duration of ICU hospitalization, time of intubation, presence and type of tracheostomy, and any subsequent reintubation. These data indicated that the median ICU and hospital stay lasted 20 (9–29) days and 28 (15–47) days, respectively. The mean intubation time was 18 days. Tracheostomy was performed in seven patients: this was surgically performed in three cases (42.8%) and by a percutaneous technique in four cases (57.1%). Symptoms of stenosis appeared between 3 and 9 weeks following ICU admission. These symptoms were inspiratory stridor, dyspnoea, persistent dry cough, wheezing, and recurrent attacks of respiratory obstruction caused by mucus; two patients (15.3%) also presented with hemoptysis. Symptoms usually occur when the tracheal diameter is reduced to 8 mm, and stridor occurs when it is less than 5 mm (12). Initially, we used awake flexible bronchoscopic examination to assess vocal fold mobility, to exclude potential airway anomalies, and to evaluate the stenosis and its main characteristics: length, location, and extent of obstruction. Tracheal stenosis can be divided into simple and complex stenosis. When the length of the stenosis segment is >1 cm, and it is accompanied by cartilage involvement, malaise, and inflammation, the stenosis is considered complex; in contrast, a stenosis segment with a length of <1 cm, with involvement limited to the mucosa and with the absence of malaise and cartilage loss, denotes “simple stenosis” (14, 15). The Myer–Cotton system of grading classifies stenosis severity on the basis of the diameter of the remaining airway in correlation with the diameter of tracheal tubes, ranging from Stage I classification for cases of less than 70% obstruction to Stage IV classification if there is 100% obstruction (16). In this study, seven patients (53.8%) presented with Stage II stenosis and six (46.2%) with Stage III. As regards the site of the stenosis, eight cases (61.5%) were located in the subglottic area and five (38.5%) in the mid-tracheal area. The diagnosis was confirmed via neck and chest CT scan with 3D reconstruction of the airways.

**Table 1:** Summary of data from 13 patients with tracheal stenosis COVID-19 related.

Data from 13 patients with tracheal stenosis									
Patient									
N°	Age	Sex	Comorbidities	Grade of stenosis	ICU stay (days)	Hospital stay (days)	Tracheostomy	Treatment	Outcome
1	49	F	Smoker, steroid	II	10	15	N	Endo	Full recovery
2	53	M	DM	III	24	31	Y	Endo (2)	Full recovery
3	72	M	DM, HTN, obesity	II	20	22	N	Endo	Full recovery
4	45	F	DM	III	23	28	Y	Endo (2)	Full recovery
5	53	M	HTN, obesity	II	22	26	Y	Endo (4)	Full recovery
6	52	F	Smoker, obesity	II	17	25	N	Endo	Full recovery
7	68	M	HTN, obesity	II	19	27	N	Endo (2)	Full recovery
8	55	F	Smoker, steroid	III	25	47	Y	Endo (4) + Surg	Full recovery
9	59	F	DM, obesity	III	23	31	Y	Endo (4) + Surg	Full recovery
10	62	F	Steroid	II	9	16	N	Endo	Full recovery
11	71	F	AMI, HTN, obesity	II	18	26	Y	Endo	Full recovery
12	48	F	Obesity	III	21	24	N	Endo (2) + Surg	Full recovery
13	57	F	Smoker, DM, obesity	III	29	46	Y	Endo (2)	Full recovery

M, male; F, female; DM, diabetes mellitus; HTN, hypertension; AMI, acute myocardial infarction.

### Endoscopic procedure

All patients underwent rigid bronchoscopy in the operating room. Patients maintained spontaneous ventilation during the entire procedure with anesthetic assistance. This treatment was facilitated by continuous propofol infusion, which enables various levels of sedation while maintaining a minimum level of discomfort. In our practice, we do not use devices such as jet ventilation or poncho; deep sedation with spontaneous breathing is therefore considered appropriate. The intervention was performed using instruments (flexible bronchoscope, dilation catheter, etc.) passed through the rigid endoscope. In cases of web-like stenosis, we applied radial incision with a pre-cut needle (Needle Knife Papillotome, 4 mm, Cook Medical). Balloon dilation (15–16.5–18 mm/3–4.5–7 ATM × 55 mm; Micro-Tech Endoscopy, Nanjing, Co. Ltd.) was introduced into the airway under direct visualization at the site of the stenosis. A stylet was used to facilitate atraumatic access across narrow stenosis. The balloon was inflated to a predetermined pressure corresponding to the desired diameter, applying controlled radial pressure to the stricture. Balloon dilation offers many advantages over the use of alternative dilatation instruments. The most important of these is that, if the balloon is placed correctly, it exerts a radial expansible force in the stenotic area and distributes this force over the entire circumference of the stenosis, avoiding rupture at any point. A diameter of 15 mm can be achieved with a pressure of 4.5 atm, and a diameter of 18 mm with 7 atm (at a length of 5.5 cm). We agreed on the three-stage technique for the sessions included in this study, in which inflation would be carried out three times, with each inflation lasting 40–60 s, according to the patient’s oxygen reserves and saturation during inflation. At the end of the session, we removed the catheter to allow ventilation and to control the result of dilation (Figure 1B). In our specialist center with a highly trained and experienced team, we recorded a negligible number of complications (dental or vocal cord trauma, hemorrhage, pneumothorax) across all such procedures conducted.

### Surgical treatment

In our institution, all patients undergoing surgical treatment (*n* = 3; 23% of the total) were treated with an anterior tracheal approach. All were positioned supine with an inflatable bag behind their shoulders and cervical extension. The incision was a classic cervical low collar incision. Initial dissection permitted us to move the upper flap to the level of the cricoid cartilage. Inferiorly, the cutaneous and platysmal flaps were raised to the sternal notch. The midline was identified and section was performed at the midline from the cricoid cartilage to the interclavicular ligament. Ligature and section of the thyroid isthmus was performed in every patient. At this time, dissection of the anterior face of the trachea was possible; this was carried out from the larynx to the carina in the anterior-lateral plane, and not in the posterior-lateral plane, to avoid injuries to the circulatory system. In every patient, a flexible endotracheal tube with its connectors and sterile anesthesia tubing was retained at the level of the incision, and the proximal anesthesia tubes were passed to the anesthesiologist. After retraction of the oro-tracheal tube and direct endoscopic control, to confirm the level of stenosis, circumferential resection of the trachea was performed (Figure 1C). Two points of PDS 3/0 were positioned at the posterior angles of the distal trachea as traction points to allow better distal dissection. In all patients, simple tracheal surgery was performed with end-to-end resection anastomosis, with posterior running suture of the membranous wall (PDS 4/0) and simple suture of the anterior wall (PDS 3/0) (Figure 1D). At the end of the surgery, two drains were left in the neck and the anterior mediastinum. All patients were immediately extubated in the operating room. In general, patients with comorbidities and poor performance status may not be eligible for surgery (17). Mortality rates of up to 5% can be seen after end-to-end anastomosis (17,18), along with complications such as restenosis, suture granuloma formation, infections, and hemorrhage (18–20). Surgical management is often definitive, but patient selection and preparation are essential for surgical success.

In the present study, no severe complications occurred during the interventional endoscopic procedures; specifically, no procedure-related deaths or immediate major complications (i.e., pneumothorax or massive bleeding) occurred, and the outcomes were uneventful in all patients. Patients reported subjective symptomatic relief immediately after the procedure, and they were able to perform normal activities and maintain normal speech. All patients underwent endoscopic treatment through rigid bronchoscopy. Stenosis recurred in eight patients (61.5%) in the period from 15 to 30 days after the first dilation procedure. All of these patients underwent a new rigid endoscopic procedure (second dilation). In two of these cases (25% of the eight patients), multiple endoscopic procedures were performed (once per month, for a total of four procedures). Three patients (two of these, 23% of the total) underwent surgical treatment as a result of multiple and/or severe recurrences. In one patient, we observed neck subcutaneous emphysema that resolved spontaneously within 72 h. The mean hospitalization time was 10.6 days (8–17). Surveillance bronchoscopy was performed at 7 and 30 days after the procedure. Final follow-up bronchoscopy demonstrated no residual stenosis and adequate respiratory space after both endoscopic and surgical treatment.

## Discussion

The treatment of iatrogenic tracheal stenosis is controversial because the role and efficacy of surgical techniques vs. endoscopic procedures depend on the experience of the staff working in a given center and on the referral pattern (20). A multidisciplinary team should plan definitive management. The characteristics of patients with this condition may make them high-risk surgical candidates; thus, endoscopic intervention is often preferable. However, all patients should be considered for surgery. Some authors have reported obtaining different results from the endoscopic procedure as an initial approach. The anatomical and functional characteristics of the laryngotracheal structure present particular difficulties. Lesions that involve the infraglottic larynx as well as the upper trachea are much more difficult to repair surgically. In our patients, we found that the tracheal cartilage healed poorly and only a limited segment of the trachea could be removed and re-anastomosis accomplished. Surgical procedures include anterior and posterior cricoid splits, mucosal and cutaneous grafts, and free grafts of cartilage and hyoid. Tracheal resection is now a well-established technique that is performed in the presence of specific indications. According to the literature, the success rate of this procedure varies from 71% to 97% (21). Complications of tracheal surgery include restenosis, dehiscence, fistula formation, and development of granulations at the suture line. Reported rates of complications are low, but this rate increases with multiple resections at increasingly high levels (22). Tracheal stenosis is evaluated on the basis of the distance from the stenotic region to the vocal cords, the length of the stenotic region, and the distance from the distal part of the stenotic region to the carina. Currently, tracheal resection is indicated in cases of high-grade mature stenosis (grades III and IV) with cranio-caudal extension >1 cm (but <5.5 cm) and/or laryngotracheal framework impairment, or in cases of a lack of response to multiple endoscopic procedures (13). Generally, patients with low-grade stenosis that is intrinsic, short (< 1 cm), and limited to a single subsite in the airway may benefit from endoscopic treatments such as radial incision or balloon dilation, alone or in combination (23). Endoscopic treatment offers minimal morbidity with good functional outcomes; however, stenosis can recur and repeated dilations may be required. This treatment is well tolerated even by heavily comorbid patients and, if adequately performed, does not cause additional injury. In contrast, indications for stent placement in cases of benign disease are controversial because of the better long-term prognosis and reported complications of stent use. In our practice, we do not use a metallic or silicone stent in benign TS. Galluccio et al. have confirmed that endoscopic procedures are a valid option in certain select cases of both simple and complex stenosis (success rate: 96% and 69%, respectively) (15). Stratakos et al. have also published analogous results (24).

In the present study, all patients were followed up for at least 12 months and achieved good clinical treatment results. Evaluation showed that after tracheal stenosis was resolved, their endoscopic condition was stable, with good mid- and long-term effects. These results suggest that the endoscopic technique is feasible, safe, and without complications. Proper selection of strategy is necessary to break the vicious cycle of “injury/recovery/stenosis/reinjury.” In cases of relapsed stenosis, the surgical procedures were definitive; no other follow-up endoscopic treatment was required.

The SARS-CoV-2 pandemic signified an unexpected challenge in terms of both the large number of patients who required ICU treatment with ventilator support and the later effects of this treatment, which included a non-negligible percentage of post-intubation TS. The increase in the use of invasive ventilation during the COVID-19 pandemic led to an overall increase in the number and severity of cases of airway damage. In Italy, the number of people with complications also increased, particularly because in northern Italy, especially in Lombardia, a large number of infections occurred during the first pandemic wave (8). Several laryngological works (25, 29) and a recent paper from the European Laryngological Society show a rise in iatrogenic sequelae (27). The incidence of and risk factors associated with tracheal stenosis in critically ill patients with COVID-19 are currently unknown, but the latter may include female sex, obesity, diabetes mellitus, cardiovascular disease, tracheostomy, prolonged intubation (including high ventilator pressure and high tube cuff pressure), and hyperinflammatory state (26). Furthermore, the need for frequent pronation cycles has been found to increase the incidence of TS. In our experience, we have recorded no differences between surgical and percutaneous tracheostomy techniques, which is in accordance with the literature (28). In post-COVID-19 patients, we expected an increased incidence of TS and have recommended that patients presenting symptoms within 6 months after ICU discharge receive a targeted evaluation aimed at ruling out the possibility of iatrogenic stenosis (29). The major limitation of our work is the small number of patients treated so far, which does not allow for a comparison with cases of non-COVID-19 TS. However, we can still confirm that endoscopic treatment is effective in patients falling into the category included in this study and can also be used as a bridge to the surgical approach.

## Data Availability

The raw data supporting the conclusions of this article will be made available by the authors without undue reservation.
